# Cryogenic‐Assisted Hydrogen Fluoride Surface Reactions Enabling Reversibly Ultra‐High Selectivity of Atomic Layer Etching Between SiO_2_ and SiN

**DOI:** 10.1002/smtd.202501744

**Published:** 2025-11-22

**Authors:** Shih‐Nan Hsiao, Makoto Sekine, Ryutaro Suda, Yoshihide Kihara, Masaru Hori

**Affiliations:** ^1^ Center for Low‐temperature Plasma Sciences Nagoya University Furocho, Chikusaku Nagoya Aichi 464‐8603 Japan; ^2^ Tokyo Electron Miyagi Ltd. Techno‐Hills, Taiwa‐cho Kurokawa‐gun Miyagi 981‐3629 Japan

**Keywords:** atomic layer etching, cryogenic, hydrogen fluoride, plasma, selectivity

## Abstract

Atomic‐level precision processes are increasingly essential for advanced semiconductor devices with highly‐complicated small features. Plasma‐enhanced atomic layer etching (ALE) is considered as a promising technique to meet requirements of material diversity and highly selective processing. Here, the ALE processes of SiO_2_ and SiN are demonstrated through manipulating hydrogen‐fluoride (HF) reactions for surface modification, followed by argon ion bombardment for material removal. By varying substrate temperature (*T*_s_) and introducing ethanol (C_2_H_5_OH) gas, which provides hydroxyl groups for cryogenic‐assisted synergistic reactions, the surface HF reactions and the properties of the surface modification layer can be significantly influenced. The etch amount per cycle (EPC) of SiN ALE decreases to zero with decreasing *T*_s_, regardless of C_2_H_5_OH addition, due to increased stability of the (NH_4_)_2_SiF_6_ modification layer. No ALE synergy is observed for SiO_2_ when C_2_H_5_OH is not added during the HF dose step, irrespective of *T*_s_. Conversely, the addition of C_2_H_5_OH at cryogenic temperatures enables the synergistic interactions between HF molecules and hydroxyl groups, enhancing the co‐adsorption of HF/C_2_H_5_OH and lowering activation energy for the fluorination reaction of the SiO_2_ that leads to the increased EPC. Consequently, the reversible etching selectivity between ALE SiO_2_ and SiN, reaching up to infinity, is achieved.

## Graphical abstract

Plasma‐enhanced atomic layer etching (ALE) of SiO_2_ and SiN is demonstrated using HF dosing for forming a modification layer and material removal by Ar ions. By controlling temperature and adding ethanol for cryogenic‐assisted reactions, surface modification and etch selectivity are tuned, achieving reversible selectivity between SiO_2_ and SiN up to infinity.

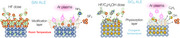

## 1 Introduction

Plasma‐assisted dry etching has been a key technology driving the evolution of the integrated circuit industry over the past five decades. In this new era, the shift from traditional semiconductor scaling methods to 3‐dimensional (3D) device architectures has transformed the field. The complexity of these advanced 3D devices intensifies the challenges faced by plasma etching, despite the fact that the certain core issues—throughput, selectivity, profile control, uniformity, defects, and damage—remain largely unchanged. Nowadays, as the critical dimensions and film thickness approach the nanometer range, atomic‐scale precision at the feature‐scale is required. Furthermore, surface roughness caused by ion bombardment during plasma etching can degrade the device reliability and performance.^[^ ^1^ ^]^ Addressing these new challenges demands advancements in plasma etching that provide new opportunities for researchers to go beyond conventional reactive ion etching.^[^ ^2^ ^]^

Digital etching or atomic layer etching (ALE), as a counterpart of atomic layer deposition (ALD), which has been widely used in the preparation of nanomaterials, has been studied for over three decades.^[^ ^3^ ^]^ ALE has been considered as a replacement for conventional steady‐state reactive ion etching in situations requiring atomic‐scale precision, ultrahigh selectivity, and atomic‐layer‐level smoothness.^[^ ^3^, ^4^ ^]^ The concept of ALE is inspired by ALD, consisting of a sequence of individual, self‐limiting surface reactions. Typically, chemical species are first introduced into the reactor, where these species react with the surface of the substrate. Ideally, only a monolayer adsorbs onto the active surface. Subsequently, energy is provided to induce chemical reactions between adsorbed species and substrate, forming volatile products. These chemical reactions can be enhanced via increasing the substrate temperature (thermal ALE), using plasma (plasma‐enhanced ALE) or employing alternative techniques like electron beams and photons.^[^ ^5^, ^6^, ^7^, ^8^ ^]^ In principle, thermal ALE is considered as an isotropic etching process and has been successfully applied to a wide range of materials.^[^ ^9^ ^]^ Plasma‐enhanced (PE) ALE, which typically employs low‐energy ions, delivers sufficient energy to remove surface atoms from materials by forming volatile products. PEALE can operate as either an anisotropic or nearly isotropic process, depending on whether a bias voltage is applied, since the ion angular distribution is closely linked to the bias voltage.^[^ ^10^ ^]^ Numerous materials have been reported using PEALE, including Si‐based dielectric films,^[^ ^11^, ^12^, ^13^, ^14^, ^15^, ^16^ ^]^ metal oxides/nitrides,^[^ ^17^, ^18^, ^19^, ^20^, ^21^, ^22^ ^]^ and metals.^[^ ^23^, ^24^ ^]^ Recently, it has also been applied to the 2D materials with low damage processing and precise thickness control.^[^ ^25^, ^26^, ^27^ ^]^

Aside from using reactive species to form a surface‐chemisorbed layer, the physisorption of neutral species on the substrate surface at cryogenic temperatures has been demonstrated as an alternative method to achieve ALE (Cryo‐ALE). Cryogenic temperatures significantly enhance the sticking possibility of the reactive species by suppressing their desorption behavior, thereby promoting physisorption and even chemisorption on the surface. The surface‐adsorbed species containing etchants can subsequently be decomposed by ion bombardment, leading to material removal. Antoun et al. explored the ALE of SiO_2_ by using physisorption of C_4_F_8_ gas and Ar sputtering at substrate temperature (*T*_s_) of −120 °C.^[^ ^28^ ^]^ Subsequently, this concept was extended to the SiN ALE using SiF_4_/O_2_ gas and Ar sputtering at *T*_s_ below −65 °C.^[^ ^29^ ^]^ The physisorption reactions of neutral gas can be achieved using high boiling point perfluorocarbons (e.g., C_5_F_8_, C_6_F_6_, C_7_F_8_), making Cryo‐ALE feasible at higher *T*_s_, ≈−20 °C.^[^ ^30^ ^]^ Recently, Adjabi et al. demonstrated the cryogenic ALE of SiO_2_ using cyclic CF_4_/Ar plasma at a *T*_S_ of −130 °C, achieving an average etch per cycle (EPC) of 0.4 nm cycle^−1^ with a process synergy of 99 %.^[^ ^31^ ^]^ Furthermore, cryogenic etch processes using hydrogen fluoride (HF)‐contained plasmas have garnered significant attention because the HF can induce additional surface reactions with SiO_2_ and SiN.^[^ ^32^, ^33^, ^34^, ^35^ ^]^ Recently, we reported the cyclic Cryo‐ALE of SiN with introducing HF gas to form a surface modification layer and using Ar sputtering for material removal.^[^ ^36^ ^]^ **Table** **1** summarizes the effects of precursors, EPC, selectivity, and *T*_s_ of the published Cryo‐ALE processes.

**Table 1:** Summary of reported cryogenic ALE processes for SiO_2_ and SiN. Some references from the same research groups employing identical methods are not included.

Material, Lead, Reference	Surface modification	Material Removal	EPC [nm/cycle]	Substrate temperature [°C]	Selectivity
Antoun et al.^[^ ^28^ ^]^	C_4_F_8_	Ar plasma	≈0.386 (SiO_2_)	−120	N.A.
Antoun et al.^[^ ^29^ ^]^	SiF_4_/O_2_ plasma	Ar plasma	≈0.675 (SiN)	−65	13.5 (SiN/p‐Si)
Sung et al.^[^ ^30^ ^]^	C_5_F_8_, C_6_F_6_, C_7_F_8_	Ar plasma	≈0.6 (SiO_2_)	−20	Up to infinite value (SiO_2_/SiN)
Adjabi et al.^[^ ^31^ ^]^	CF_4_/Ar plasma	Ar plasma	≈0.4 (SiO_2_)	−130	N.A.
Hsiao et al.^[^ ^36^ ^]^	HF	Ar plasma	≈0.2 (SiN)	−30	N.A.
This work	HF only HF/C_2_H_5_OH	Ar plasma	≈3.1 (SiN) ≈0.79 (SiO_2_)	20– −60	12 (SiN/SiO_2_) Up to infinite value (SiO_2_/SiN)

**Figure 1:**
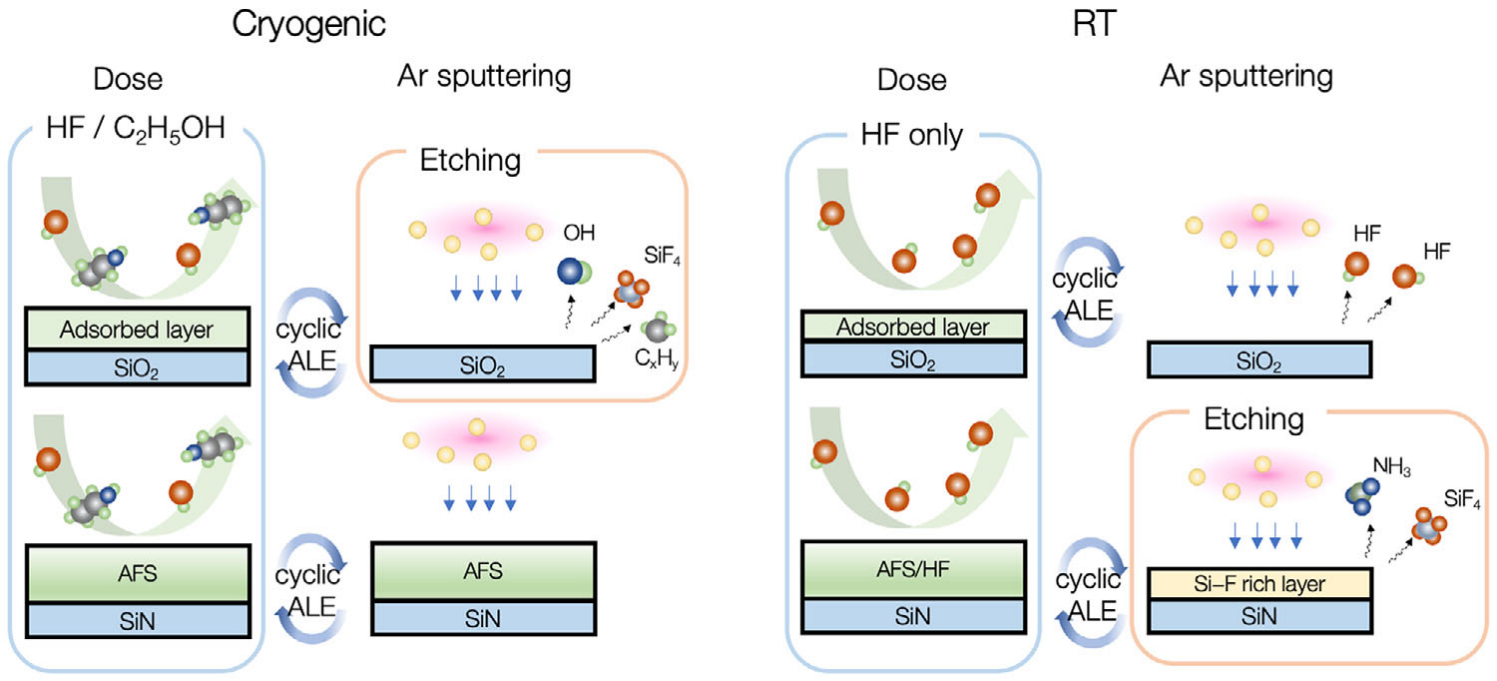
Overview of ALE of SiO_2_ and SiN at cryogenic and room temperature.

This study outlines the ALE process with etching selectivity controlled by chemical reactions between HF gas and material surfaces. The ALE of SiO_2_ and SiN of alternating HF dose and Ar plasma exposure is investigated, as illustrated in **Figure** **1**. By adjusting the *T*_s_ and introducing ethanol (C_2_H_5_OH) as an additive to HF gas during the surface modification step, the etching selectivity between SiO_2_ and SiN can be turned to favor SiO_2_ or SiN, achieving effectively infinite selectivity under certain conditions. The surface reaction mechanism for the SiO_2_ and SiN films during ALE was clarified using in situ attenuated total reflection Fourier transformation infrared spectroscopy (ATR‐FTIR). The analysis revealed the formation of ammonia fluorosilicate (AFS) on the SiN surface during HF dosing, regardless of C_2_H_5_OH addition or *T*_s_. The ALE can be achieved at *T*_s_ ≥ −30 °C but ceased at lower temperatures, depending on whether the AFS layer can be removed by the Ar ion bombardment. On the other hand, the C_2_H_5_OH was found to act as a catalyst for SiO_2_ etching through HF physisorption and Ar plasma exposure. The etch depth per cycle (etching selectivity of SiO_2_/SN) increased from ≈0.29 to 0.79 nm cycle^−1^ (0.87 to an effectively infinite value) as the *T*_s_ decreased from 20 to −60 °C, due to the higher accumulation of HF/C_2_H_5_OH on the surface. The effect of C_2_H_5_OH on SiN etching exhibited a reverse trend compared to that on SiO_2_, implying that although the etching process in both materials proceeds via a common HF physisorption–assisted mechanism involving C_2_H_5_OH, the inherent differences in their surface chemistries lead to divergent etching outcomes.

## 2 Experimental Section

**Figure 2:**
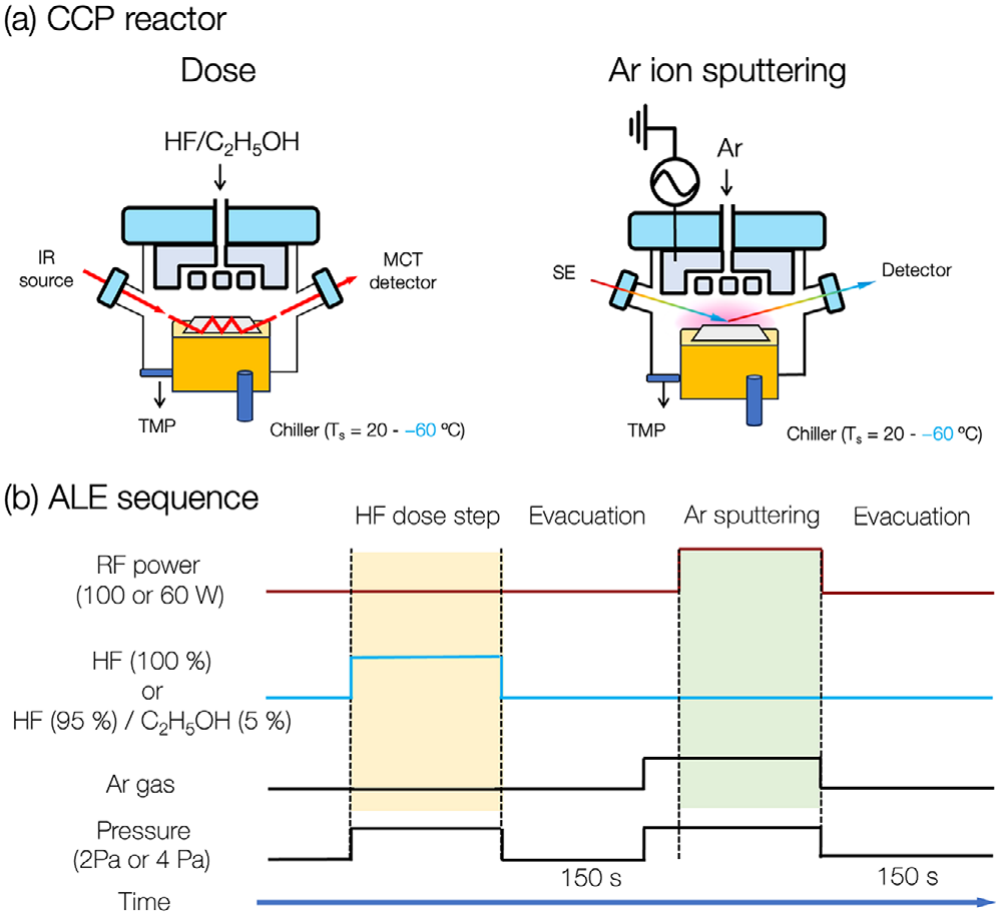
a) Schematic illustration of the custom‐designed rector for the ALE experiments. b) Parameter sequence in the ALE process. The parameters with RF power of 100 W (60 W), pure HF dosing (HF with 5 % of C_2_H_5_OH), and pressure at 2 Pa (4 Pa) were used in experiments presented in Sections 3.1 and 3.2. The bias power was set to 0 W (the substrate remained at a floating potential).

A custom‐made reactor with a capacitively‐coupled plasma source was used for the ALE in this work, as illustrated in **Figure** **2**a. The vacuum condition was maintained at a pressure better than 7 × 10^−5^ Pa before experiments were conducted. The SiO_2_ and SiN films were grown on Si wafers or Ge prims (for ATR‐FTIR experiments) using plasma‐enhanced chemical vapor deposition. Further details regarding the properties of the SiO_2_ and SiN films are summarized in **Table** **2**. These films were characterized using ellipsometry, x‐ray reflectivity, and Rutherford backscattering spectroscopy, as described in our previous publication.^[^ ^37^ ^]^ The sample was placed on a 4‐inch Si carrier wafer using a fluorinated grease, which offers high stability at temperatures as low as −70 °C, to improve thermal conductivity between the carrier wafer and the sample. The carrier wafer was fixed using an electrostatic chuck. The *T*_s_ was controlled by a circulated coolant system, ranging from 20 to −60 °C. A helium back flowing with a pressure higher than 700 Pa was introduced to improve the thermal conductivity. For the cyclic ALE processes, as shown in Figure 2b, HF gas, with or without the addition of 5 % C_2_H_5_OH (by flowing rate), is introduced for surface modification through a showerhead top electrode, followed by an Ar plasma exposure for material removal. The top electrode was operated at a frequency of 100 MHz for the Ar plasma discharge. The power input and the pressure of the Ar plasma were fixed at 100 W (60 W) and 2 Pa (4 Pa) for experiments shown in Sections 3.1 and 3.2, respectively, while the substrate was not biased and kept at the floating potential.

**Table 2:** Deposition method, thickness, mass density, refractive index, and chemical composition of the SiN and SiO_2_ films before the ALE process.

	Deposition method	Thickness [nm]	Density [g cm^−3^]	Refractive index at 633 nm	Composition
SiN	PECVD	≈30	2.24 ± 0.04	1.683	Si_39.2_N_41.3_H_19.5_ (± 0.2 at %)
SiO_2_	PECVD	≈30	2.40 ± 0.06	1.369	Si_28.9_O_59.2_H_12.0_ (± 0.2 at %)

The thickness variation during the ALE processes was analyzed with in situ spectroscopic ellipsometer (SE) system equipped with a Xe light source with a multi‐wavelength range from 200 to 1000 nm (J.A. Woollam Co. 2000 M). The optical model employed for the SE analyses was based on the Tauc–Lorentz oscillators, further calibrated using the actual film thickness measured by X‐ray reflectometry (Rigaku Co. SmartLab).^[^ ^37^ ^]^ The surface structure changes during the ALE process were analyzed with in situ ATR‐FTIR, as shown in Figure 2a. The experiments were conducted on the Ge prism samples with the FTIR system (Thermo Fisher Scientific, iS50). The transmitted IR signal through the Ge prism and KBr windows was acquired with a mercury cadmium telluride (MCT) detector. The acquired spectra spanned a wavenumber range of 700–4000 cm^−1^ with a resolution of 4 cm^−1^. To ensure a high signal‐to‐noise ratio, each spectrum presented in this work was obtained by averaging at least 128 scans. The Ar^+^ ion energy distribution was measured with a commercial retarding field energy analyzer (RFEA, Impedans Ltd., Semion System 500). The analyzer was placed on a Si carrier wafer without applying a bias voltage. The ion energy distribution function of the Ar plasma is illustrated in Figure S1 (Supporting information). The peak of the ion energy distribution spectrum was found to be ≈20–22 eV.

## 3 Results and Discussion

### 3.1 ALE Process Without C_2_H_5_OH Additive

**Figure 3:**
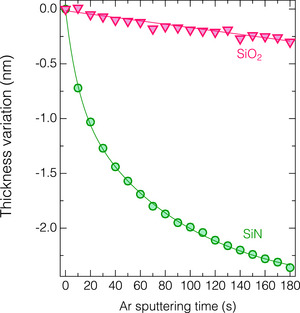
In situ thickness variation of SiO_2_ and SiN as a function of the Ar sputtering time at *T*_s_ of 0 °C.

As presented in our previous publications, anhydrous HF can react with a damaged or a highly‐hydrogenated SiN surface to form an AFS layer.^[^ ^36^, ^38^ ^]^ The formation of the AFS layer plays a salient role in ALE by enabling self‐limiting etch reactions, which have also been reported in other plasma systems.^[^ ^39^, ^40^ ^]^ Conversely, anhydrous HF molecules do not function as an etchant for SiO_2_ under low‐pressure conditions without any catalytic assistance. These phenomena are likewise observed in this study, as shown in **Figure** **3**. Figure 3 compares the ALE process of SiO_2_ and SiN under Ar plasma exposure following an anhydrous HF dose at *T*_s_ = 0 °C. The ALE of SiN demonstrates a typical self‐limiting etch reaction, characterized by a rapid initial decrease in relative thickness decreased quickly at the onset of the Ar sputtering, followed by a linear decrease over time due to physical sputtering. Conversely, for the SiO_2_ sample, the relative thickness decreased monotonically with increasing Ar exposure time, indicating the absence of any ALE synergistic reactions.^[^ ^41^ ^]^ One may notice that the steady‐state etch rate observed in the SiN (as the Ar sputtering time exceeds ≈120 s) was higher than that in the SiO_2_. This can be explained by the higher dissociation energy of Si─O (6.15 eV) than that of Si─N (4.77 eV).^[^ ^42^, ^43^ ^]^

**Figure 4:**
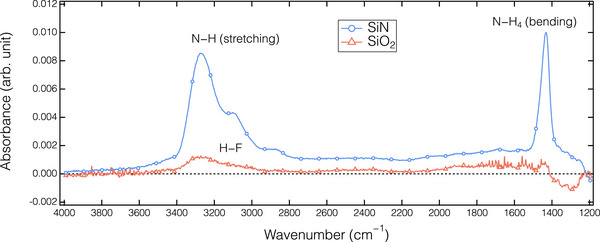
Absorbance spectra of the SiN and SiO_2_ after a half‐cycle of the ALE process at *T*_s_ = 20 °C.

The discrepancy in surface structure between SiN and SiO_2_ after the HF dosing step (a state before Ar plasma exposure in Figure 3) was analyzed using in situ FTIR analysis. **Figure** **4** illustrates the absorbance changes in the SiN and SiO_2_ films after introducing HF gas for surface modification. In the SiN spectrum, the positive peaks observed at ≈3250 and 1430 cm^−1^ correspond to the formation of N─H stretching and N─H_4_ bending vibration bands, respectively, indicating the formation of the AFS phase.^[^ ^36^, ^38^ ^]^ On the other hand, the physisorption of HF molecules on the SiO_2_ surface was identified by a weak IR adsorption peak at ≈3300 cm^−1^. However, the physisorbed HF layer did not assist the etching during the Ar plasma exposure. The detailed mechanism will be discussed later.

**Figure 5:**
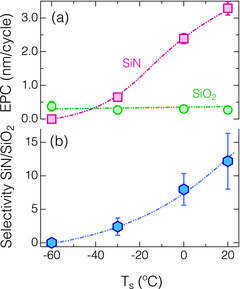
Dependence of (a) etch amount per cycle (EPC) of the SiN and SiO_2_ ALE and (b) etching selectivity of SiN against SiO_2_ on the substrate temperature (*T*_s_).

**Figure** **5** presents the dependences of EPC and etch selectivity of SiN/SiO_2_ on *T*_s_ under Ar plasma conditions, as shown in Figure 3. As illustrated in Figure 5a, the EPC for SiN declined significantly from 3.1 nm cycle^−1^ to zero (no etching) as *T*_s_ decreased from 20 to −60 °C, whereas the EPC for SiO_2_ remained nearly a constant value at ≈0.3 nm cycle^−1^.　For the SiN ALE case, the decrease in EPC was attributed to the higher stability of the AFS layer and the increased amount of surface‐absorbed species, as discussed in the previous publication.^[^ ^36^ ^]^ A similar trend of decreased ER with decreasing *T*_s_ was also observed in the SiN with RIE using HF‐contained plasmas.^[^ ^32^, ^34^ ^]^ Conversely, the EPC of SiO_2_ remined unaffected by *T*_s_, due to the absence of ALE synergy, with only Ar physical sputtering for the films, as shown in Figure S2 (Supporting information). The in situ FTIR results reveal that the absorbance intensity of the H─F bands during HF dose step increased with decreasing *T*_s_, as illustrated in Figure S3 (Supporting information), indicating an increase in the amount of physisorbed HF layer. The observed physisorption of HF on the SiO_2_ aligns with the computational findings reported by Chowdhury et al.^[^ ^44^ ^]^ However, the physisorbed HF layer did not assist the etching during the Ar plasma exposure, which contrasts with the findings from the RIE studies with HF‐contained plasmas at cryogenic temperatures.^[^ ^34^, ^35^, ^45^ ^]^ This suggests the critical role of H_2_O as a catalyst for the surface catalytic reactions, particularly under the conditions involving Ar plasma with low‐energy ions. Given that the bonding energies of H─F (≈6.0 eV)^[^ ^42^ ^]^ are significantly higher than that of Si─O (≈4.1 eV),^[^ ^46^ ^]^ and the physisorption energy of HF molecules is relatively low (≈0.5 eV),^[^ ^44^ ^]^ Ar ions merely vaporize the physisorbed HF layer without breaking the H─F bonds, consistent with our previous findings.^[^ ^47^ ^]^

### 3.2 ALE Process with C_2_H_5_OH Additive

**Figure 6:**
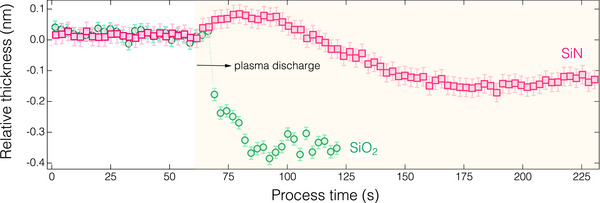
In situ thickness variation of the ALE (with C_2_H_5_OH addition during HF dosing) of SiN and SiO_2_ at *T*_s_ = −20 °C.

**Figure 7:**
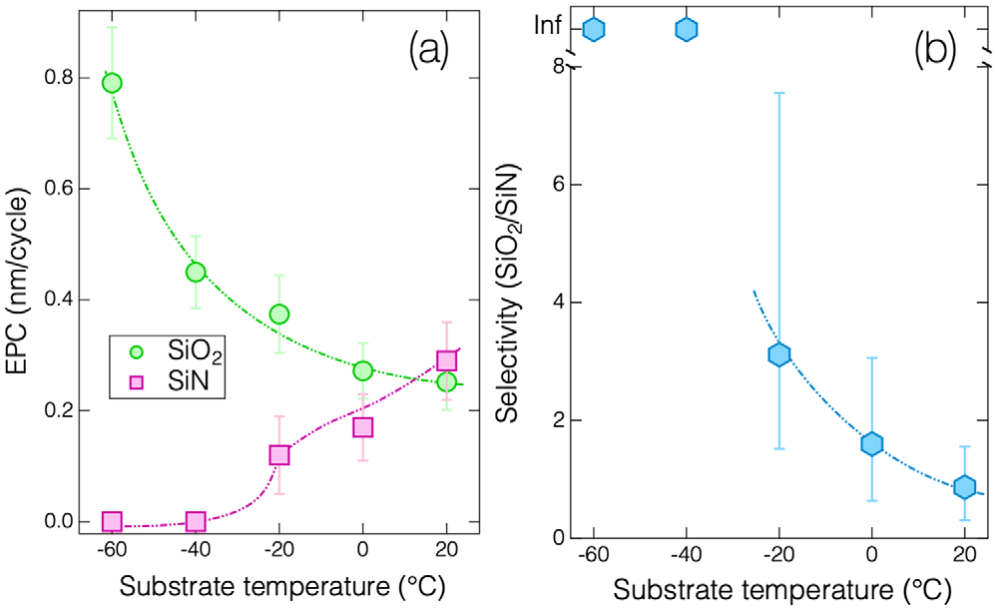
Dependence of (a) etch amount per cycle (EPC) of the SiN and SiO_2_ ALE and (b) etching selectivity of SiO_2_ against SiN on the substrate temperature (*T*_s_). The “Inf” on the vertical axis indicates an effectively infinite etching selectivity.

Surface hydroxyl groups in SiO_2_ have been demonstrated to exhibit a catalytic effect on the surface reactions for fluorination of SiO_2_ using the density functional theory calculations.^[^ ^48^ ^]^ Our previous findings also showed that the co‐adsorption of HF and H_2_O at the cryogenic temperature significantly enhances the etching throughput of SiO_2_.^[^ ^45^, ^47^ ^]^ A similar concept has been applied for isotropic etching of SiO_2_ through interactions between HF and methanol gases,^[^ ^49^ ^]^ as an alternative to H_2_O. In this section, 5% of ethanol (C_2_H_5_OH) was introduced during the HF dosing step to modify the surface reactions on the substrate. The primary rationale for selecting ethanol as the additive instead of H_2_O was to potentially achieve selectivity between SiO_2_ and SiN, based on the findings of Lill et al.,^[^ ^34^ ^]^ which demonstrated that the presence of H_2_O can reduce the bonding energy within the AFS phase, thereby weakening its stability when NH_4_F in AFS is replaced by H_2_O. **Figure** **6** illustrates the representative feature of SiO_2_ and SiN ALE during Ar plasma exposure at *T*_s_ = −20 °C, demonstrating self‐limiting etch reaction for both films, with EPC values of ≈0.38 for SiO_2_ and 0.12 nm cycle^−1^ for SiN, respectively. **Figure** **7**a shows that the EPC for SiO_2_ ALE increased from 0.25 to 0.79 nm cycle^−1^ with decreasing *T*_s_ from 20 to −60 °C, whereas the EPC of the SiN ALE declined from 0.29 nm cycle^−1^ to zero. The variation in EPC for SiO_2_ and SiN with *T*_s_ resulted in an increase in etch selectivity from 0.87 to effectively infinite as *T*_s_ ≤ −40 °C, as illustrated in Figure 7b. The results highlight that ultra‐high etching selectivity can be achieved through self‐limiting reactions facilitated by cryogenic‐assisted surface HF interactions, which will be discussed later.

**Figure 8:**
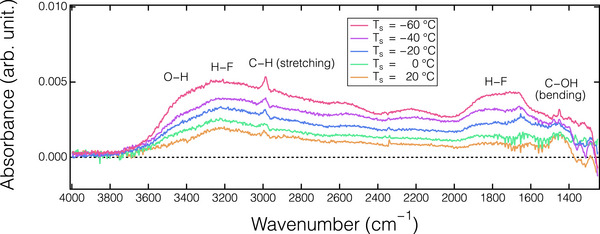
Absorption spectra of SiO_2_ film after dosing HF/C_2_H_5_OH of the ALE process at different substrate temperatures (*T*_s_).

**Figure** **8** presents the absorbance spectra observed for the SiO_2_, as analyzed with the in situ ATR‐FTIR, after introducing HF/C_2_H_5_OH gas during an ALE cycle at different *T*_s_. Note that the reference spectrum for these absorbance spectra was acquired from the thin films prior to the HF/C_2_H_5_OH dosing. A broad positive band ranging from 3500 to 1500 cm^−1^ is observed. By comparing the spectra with that of physisorbed HF without an additive, as illustrated in Figure 4, the O─H band at ≈3500 cm^−1^, the C─H peak at ≈2980 cm^−1^, and the C─OH band at 1480 cm^−1^, corresponding to the presence of C_2_H_5_OH, were identified.^[^ ^50^ ^]^ This suggests the co‐adsorption of HF/C_2_H_5_OH on the SiO_2_ surface after the dosing half‐cycle. Additionally, the absorbance intensity significantly increased as *T*_s_ decreased, indicating a higher amount of the co‐adsorbed HF/C_2_H_5_OH on the cooled SiO_2_ surface. In vapor etching of SiO_2_ using HF gas, the addition of H_2_O, methanol (CH_3_OH), and C_2_H_5_OH, each containing hydroxyl bonds, has been demonstrated to enhance ER.^[^ ^51^, ^52^, ^53^ ^]^ This enhancement is attributed to the catalytic role of hydroxyl groups involved in the etching step, which significantly lower the energy barrier for SiO_2_ etching.^[^ ^48^, ^54^ ^]^ More importantly, the ER increases as *T*_s_ decreases, presumably due to a higher converge of gas molecules on the cooled substrate surface, as predicted by Langmuir theory.^[^ ^49^, ^53^ ^]^ Our experimental observations, with in situ ATR‐FTIR, also support the proposed mechanism.

**Figure 9:**
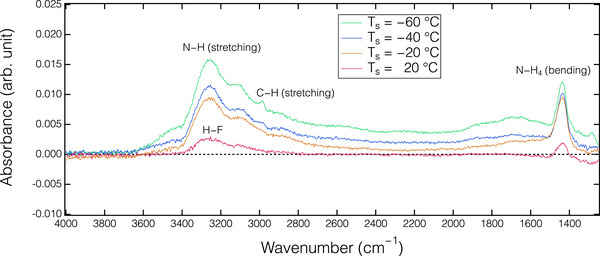
Absorption spectra of SiN film after dosing HF/C_2_H_5_OH of the ALE process at different substrate temperatures (*T*_s_).

The absorbance spectra of the SiN films after the HF/C_2_H_5_OH dosing step of an ALE cycle at various *T*_s_ are presented in **Figure** **9**. The spectra indicate the formation of the AFS phase after the dosing step half‐cycle, resembling to the results obtained without adding C_2_H_5_OH gas, as shown in Figure 4. Comparing the spectrum obtained without the addition of C_2_H_5_OH gas (also see Figure S3, Supporting information), the absorbance intensity of the N─H stretching and N─H_4_ bending vibration bands significantly decreased when C_2_H_5_OH was introduced during HF dosing. This suggests that even a 5 % addition of C_2_H_5_OH can significantly inhibit the formation of the AFS phase on the SiN. While the detailed suppression mechanism remains unclear, a plausible explanation is that the hydroxyl groups in the C_2_H_5_OH may attract the HF molecules. Consequently, the limited availability of HF to react with −NH_x_ bonds in SiN reduces the formation of the AFS phase, leading to a lower EPC value in the ALE process. Further experiments and numerical modeling of the chemical structure are required to elucidate the behavior and underlying mechanism of the AFS suppression. As demonstrated by both experimental and modeling studies, AFS is known unstable at elevated temperatures, but resists decomposition under cryogenic conditions.^[^ ^40^, ^43^, ^55^ ^]^ Based on the RFEA measurement, the obtained peak of the ion energy spectrum was ≈22 eV, which is close to the sputtering ion threshold of SiN (≈25 eV).^[^ ^56^, ^57^ ^]^ When the substrate is cooled, the ion energy threshold required for dissociation of the AFS and other chemisorbed species increases, resulting in the cessation of etching at *T*_s_ ≤ −40 °C.^[^ ^36^, ^58^ ^]^

### 3.3 Overall Etching Mechanism with Different Surface HF Reactions

**Figure 10:**
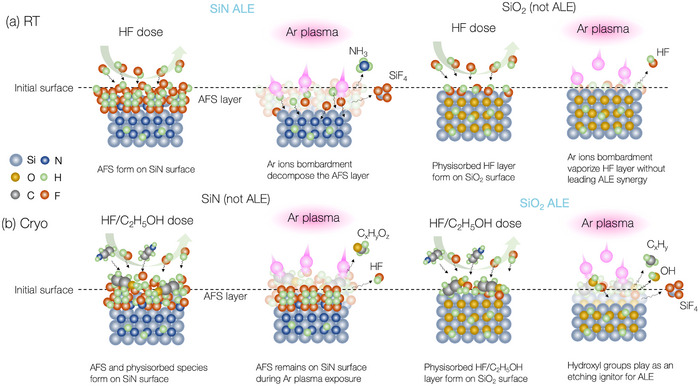
Schematic of the etching mechanism for the present ALE process.

The results from in situ characterizations highlight the benefits from ALE combined with cryogenic techniques, which enable ultra‐high etching selectivity by controlling surface HF reactions across the SiN and SiO_2_. **Figure** **10** shows the schematic for the proposed ALE reaction mechanism and its impact on overall selectivity. When the ALE process is performed at room temperature, the AFS forms spontaneously via the HF reactions with SiN during the dosing step.^[^ ^38^ ^]^ Subsequently, Ar ions are introduced to dissociate the surface modification layer, as presented in our early publication.^[^ ^36^ ^]^ Conversely, HF can only form a physisorption layer on the SiO_2_ surface. Without the assistance of hydroxyl groups, the physisorbed HF layer alone does not result in an ALE synergy during Ar ion bombardment. Consequently, an etching selectivity of SiN/SiO_2_ can be achieved. Due to the physical sputtering of SiO_2_ upon Ar ion bombardment, our results demonstrate an etching selectivity of only ≈12. Because it is well known that SiO_2_ exhibits a higher sputtering threshold energy (≈45 eV)^[^ ^59^ ^]^ than SiN (≈25 eV)^[^ ^56^, ^60^ ^]^ under Ar plasma, an effectively infinite etching selectivity of SiN over SiO_2_ etching could be expected if ion energy is carefully controlled. The formation of the AFS phase in the first half‐cycle reaction of the SiN ALE can be expressed as:

(1) $${\mathrm{Si}}_{3}N_{4\left(s\right)}+16{\mathrm{HF}}_{\left(g\right)}\;\to \;2{\left({\mathrm{NH}}_{4}\right)}_{2}{\mathrm{SiF}}_{6\left(s\right)}+{\mathrm{SiF}}_{4\left(g\right)}$$

Here, the stoichiometric composition of SiN is used to represent the general case. However, the presence of hydrogen impurities in the SiN film significantly reduces the activation barrier for the formation of the AFS phase, as a spontaneous reaction can occur through the chemisorption of HF molecules onto the N─H bonds in the SiN substrate.^[^ ^38^, ^43^ ^]^ When AFS is bombarded by Ar with sufficient energy during the second half‐cycle, AFS decomposes into volatile byproducts.

(2) $$2{\left({\mathrm{NH}}_{4}\right)}_{2}{\mathrm{SiF}}_{6\left(s\right)}+\mathrm{ions}\;\to \;2{\mathrm{NH}}_{3\left(g\right)}+{\mathrm{SiF}}_{4\left(g\right)}+2{\mathrm{HF}}_{\left(s\right)}$$

Again, the stoichiometric composition of these compounds is presented. However, energetic ions typically decompose the AFS (even when it is off‐stoichiometric) into smaller fragments, such as SiF*_x_* (*x* = 1–3), NH*_x_* (*x* = 1–2) or even other hydrogen‐containing compounds such as SiH*_x_* F*_y_* (*x* + *y*  ≤ 4).^[^ ^43^, ^61^ ^]^ The decomposition step could also be established by using heat or other energetic species.^[^ ^34^, ^39^, ^40^ ^]^

As illustrated in Figure 10b, the ALE process with the addition of C_2_H_5_OH in HF during the dosing step is conducted at cryogenic temperatures. During the dosing step, the AFS forms on the SiN surface at low temperature via the spontaneous HF reaction, although the formation is suppressed due to the presence of C_2_H_5_OH in the co‐adsorption layer of HF/C_2_H_5_OH on the SiN surface. Following that, Ar ions cannot dissociate the AFS because of its increased stability at cryogenic temperatures.^[^ ^36^, ^43^ ^]^ Similarly, a co‐adsorption layer of HF/C_2_H_5_OH forms on the SiO_2_ surface. The hydroxyl groups in C_2_H_5_OH act as a catalyst for the dissociation of H─F and reduce the activation energy of SiO_2_ fluorination, significantly enhancing SiO_2_ ALE. This process is further enhanced at cryogenic temperatures due to the increased amount of HF/C_2_H_5_OH co‐adsorption layer. The adsorption reaction during the dosing step can be expressed as:

(3) $${\mathrm{SiO}}_{2(s)}\;+\;{\mathrm{HF}}_{\left(g\right)}+C_{2}H_{5}{\mathrm{OH}}_{\left(g\right)}\;\to \;{\mathrm{SiO}}_{2(s)}+\mathrm{HF}/C_{2}H_{5}{\mathrm{OH}}_{\left(\mathrm{ad}.\right)}$$

The co‐adsorption of HF and C_2_H_5_OH occurs because both HF and hydroxyl groups can form strong hydrogen‐bonding networks, although HF forms particularly strong bonds due to the high electronegativity of fluorine.^[^ ^42^ ^]^ However, we assume that the hydrogen‐bonded network layer is more likely to correspond to monolayer adsorption, that is, Langmuir‐type adsorption, as discussed in Section 3.2, which warrants further detailed investigation. Subsequently, Ar ions induce an etching reaction, assisted by the catalytic reactions between hydroxyl groups and HF molecules:^[^ ^48^ ^]^

(4) $$\begin{array}{ccc} & & {\mathrm{SiO}}_{2(s)}\;+\;4\mathrm{HF}/C_{2}H_{5}{\mathrm{OH}}_{\left(\mathrm{ad}.\right)}+\mathrm{ions}\;\to \\ & & \;{\mathrm{SiF}}_{4(g)}+2H_{2}O_{\left(g\right)}+{\mathrm{CH}}_{x(g)}+{\mathrm{OH}}_{\left(g\right)} \end{array}$$

The reaction of SiO_2_ with HF generates volatile byproducts of SiF_4_ and H_2_O via a pseudo‐ etching mechanism has been demonstrated in a mass‐production etcher using quadrupole mass spectroscopy and optical emission spectroscopy.^[^ ^33^ ^]^ Consequently, at cryogenic temperatures, the surface reactions involving HF enhance the ALE of SiO_2_ through the catalytic reactions while hindering SiN etching by stabilizing AFS against etching, thereby enabling ultra‐high etching selectivity. The impact of C_2_H_5_OH on SiN etching was found to be opposite to its effect on SiO_2_, indicating that the same fundamental reaction pathway—mediated by HF physisorption and surface interactions with C_2_H_5_OH—operates in both systems. However, the distinct surface chemistries of SiN and SiO_2_ lead to markedly different etching behaviors, resulting in the suppression of SiN etching while promoting SiO_2_ removal under identical processing conditions.

## 4 Conclusion

This study demonstrates the tunable etching selectivity of atomic layer etching (ALE) across SiO_2_ and SiN films by manipulating the cryogenic‐induced surface HF reactions and Ar plasmas. By using the in situ monitoring techniques, including spectroscopic ellipsometry and attenuated total reflection FTIR, we found that the surface reactions and the ALE characteristics are strongly influenced by the interplay of additives, material properties, and substrate temperature (*T*_s_). At room temperature, HF molecules react spontaneously with SiN to form (NH_4_)_2_SiF_6_ (AFS) phase during the HF dose step, which is subsequently removed by Ar ion bombardment. In contrast, no ALE synergy occurs for the SiO_2_ with the same conditions, resulting in a potentially infinite selectivity of SiN over SiO_2_ (≈12 was obtained due to physical sputtering of the SiO_2_). On the other hand, at cryogenic temperatures, the addition of C_2_H_5_OH gas during the HF dosing step, with its hydroxyl groups acting as a catalyst, was found to induce ALE synergy for the SiO_2_. The etch depth per cycle increased because of the enhanced accumulation of the co‐adsorbed HF/C_2_H_5_OH layer as *T*_s_ decreased. At *T*_s_ ≤ −40 °C, the AFS phase on the SiN solidifies and becomes immovable by the Ar ions, resulting an effectively infinite selectivity of SiO_2_ over SiN. Our findings demonstrated the benefits of ALE combined with cryogenic‐assisted surface reactions, enabling tunable ultrahigh materials etching selectivity for potential applications in advanced nanostructured semiconductor devices.^[^ ^62^ ^]^

## Conflict of Interest

The authors declare no conflict of interest.

## Supporting information



Supporting Information

## Data Availability

The data that support the findings of this study are available from the corresponding author upon reasonable request.
