# Supplementary material for: Cryogenic‐Assisted Hydrogen Fluoride Surface Reactions Enabling Reversibly Ultra‐High Selectivity of Atomic Layer Etching Between SiO2 and SiN
Source: Small Methods. 2025 Nov 22;10(1):e01744. doi: 10.1002/smtd.202501744 (PMC12790377; doi:10.1002/smtd.202501744)
Supplement: Supplementary file 1 — Supporting Information [file SMTD-10-e01744-s001.docx]

*Supporting Information*

Cryogenic-Assisted Hydrogen Fluoride Surface Reactions Enabling Reversibly Ultra-High Selectivity of Atomic Layer Etching between SiO_2_ and SiN

Shih-Nan Hsiao^1*^, Makoto Sekine^1^, Ryutaro Suda^2^, Yoshihide Kihara^2^, and Masaru Hori^1^

^1^ Center for Low-temperature Plasma Sciences, Nagoya University, Furocho, Chikusaku, Nagoya, Aichi 464-8603, Japan

^2^ Tokyo Electron Miyagi Ltd., Techno-Hills, Taiwa-cho, Kurokawa-gun, Miyagi, 981-3629, Japan

*Corresponding authors: Shih-Nan Hsiao (hsiao.shih.nan.t8@f.mail.nagoya-u.ac.jp)

Figure S1: Ion energy distribution functions measured using a retarding field energy analyzer (RFEA) during the Ar plasma step of the ALE process under various RF power inputs.

Figure S2: The relative thickness change, obtained by in situ SE, of the SiO_2_ (ALE process with pure HF dosing) as a function of Ar sputtering time at different substrate temperatures.

Figure S3: FTIR spectra of the SiO_2_ films during the HF dosing at *T*_s_ = −60 and 20 °C.
